# Dr Neal and the Poor-Law Service

**Published:** 1913-09-20

**Authors:** 


### DR NEAL AND THE POOR-LAW SERVICE.

### PRESENTATION AT ST. JOHN'S HILL INFIRMARY.

A large and enthusiastic gathering of past and present officers of the Wandsworth Union took place on Wednesday, September 10, at the Board Room of the Wandsworth Guardians, to present Dr. J. Breward Neal, late medical superintendent of the infirmary, with a silver tea service, silver rose bowl, silver cake basket, settee, and reading stand as an expression of esteem.

Mr. Piper, clerk to the Guardians, was in the chair, and referred to the long period of office Dr. Neal had served—viz. 34½ years. He also spoke with appreciation of the interest Mrs. Neal had taken in the welfare of the institution. Dr. Maccormac, Dr. Neal's successor, in a happy speech, expressed the feelings of the many officers who had received sympathetic and practical help in their work.

Dr. Neal thanked the gathering for their handsome presents, and spoke of the regret he felt in severing his connection with the Poor-Law Service. He mentioned that no less than 103,000 patients had been admitted during the time he had been superintendent, and drew attention to the great amount of work this had involved and to the assistance of all the officers who had loyally worked with him.

